# Biomarkers for Detecting Kidney Dysfunction in Type-2 Diabetics and Diabetic Nephropathy Subjects: A Case-Control Study to Identify Potential Biomarkers of DN to Stratify Risk of Progression in T2D Patients

**DOI:** 10.3389/fendo.2022.887237

**Published:** 2022-06-29

**Authors:** Carla Harkin, Diego Cobice, Simon Brockbank, Stephanie Bolton, Frances Johnston, Anna Strzelecka, Joanne Watt, Mary Jo Kurth, John V. Lamont, Peter Fitzgerald, Tara Moore, Mark W. Ruddock

**Affiliations:** ^1^ Biomedical Sciences Research Institute, Ulster University, Coleraine, United Kingdom; ^2^ Randox Laboratories Ltd, Clinical Studies Group, Randox Science Park, Antrim, United Kingdom; ^3^ Renal Unit, Antrim Area Hospital, Antrim, United Kingdom; ^4^ Diabetes Clinic, Whiteabbey Hospital, Newtownabbey, United Kingdom

**Keywords:** diabetic nephropathy, midkine, sTNFR1, sTNFR2, L-FABP, H-FABP, cystatin C, type-2 diabetes

## Abstract

**Introduction:**

Currently there are no biomarkers that are predictive of when patients with type-2 diabetes (T2D) will progress to more serious kidney disease i.e., diabetic nephropathy (DN). Biomarkers that could identify patients at risk of progression would allow earlier, more aggressive treatment intervention and management, reducing patient morbidity and mortality.

**Materials and Methods:**

Study participants (N=88; control n=26; T2D n=32; DN n=30) were recruited from the renal unit at Antrim Area Hospital, Antrim, UK; Whiteabbey Hospital Diabetic Clinic, Newtownabbey, UK; Ulster University (UU), Belfast, UK; and the University of the Third Age (U3A), Belfast, UK; between 2019 and 2020. Venous blood and urine were collected with a detailed clinical history for each study participant.

**Results:**

In total, 13/25 (52.0%) biomarkers measured in urine and 25/34 (73.5%) biomarkers measured in serum were identified as significantly different between control, T2D and DN participants. DN patients, were older, smoked more, had higher systolic blood pressure and higher serum creatinine levels and lower eGFR function. Serum biomarkers significantly inversely correlated with eGFR.

**Conclusion:**

This pilot-study identified several serum biomarkers that could be used to predict progression of T2D to more serious kidney disease: namely, midkine, sTNFR1 and 2, H-FABP and Cystatin C. Our results warrant confirmation in a longitudinal study using a larger patient cohort.

## Introduction

Almost all patients with diabetes will display some evidence of kidney disease (1, 2). However, it can take several years for kidney damage to progress and symptoms may only become apparent when significant damage to the kidney has occurred. Indeed 20 to 40% of diabetic patients will progress to more serious kidney disease e.g. diabetic nephropathy (DN), also called diabetic kidney disease (DKD), chronic kidney disease (CKD), or kidney disease of diabetes (3).

DN is a serious kidney-related complication of diabetes and is one of the leading causes of end-stage renal disease (ESRD) requiring extensive patient management (4). The average patient cost for dialysis is £30,800/year. Kidney transplantation costs £17,000/patient and the immunosuppression drug regime required for each transplant recipient is a further £5,000/year.

Almost 1 in 10 adults in the UK are affected by CKD, costing the NHS over £1.5 billion/year (5). Kidney disease costs the NHS more than breast, lung, colon and skin cancer combined, yet many cases remain undiagnosed and untreated, according to a recent report by NHS Kidney Care (6). Similarly, almost 12.5% of the US population show evidence of CKD (7, 8). However, patient awareness is low, and presentation is late (CKD stages 4/5). As a result, the Medicare expenditure for all patients who were diagnosed with CKD exceeded $81.8 billion in 2018 (9), up 23% from 2015 (10). Furthermore, Medicare expenditure for patients with CKD aged 65 and older exceeded $67 billion, which represented almost 25% of all Medicare spending for this age group (11).

Currently, diabetic patients are routinely monitored by measuring urinary protein (proteinuria) and serum creatinine (12). However, albuminuria rather than proteinuria has proven more clinically meaningful in patients with DN (13).

Proteinuria is usually divided into three categories: transient or intermittent, orthostatic and persistent. Transient proteinuria is the most commonly reported form of proteinuria. Elevated proteinuria levels reflect the extent of the damage to the glomeruli whereas serum creatinine is used to calculate the estimated glomerular filtration rate (eGFR) (14). Albuminuria is an early indicator of DN that is used to predict progression of ESRD (15). Furthermore, increased albuminuria is also a risk factor for cardiovascular morbidity and mortality in diabetic patients (16).

Clinical risk factors for DN progression include age, BMI, smoking status, glycated haemoglobin (HbA1c), elevated systolic blood pressure, diabetic retinopathy, high-density lipoprotein, triglyceride, increased urinary albumin excretion and declining eGFR (17–19). Patients identified at risk of DN, or progression, are normally prescribed angiotensin converting enzyme (ACE) inhibitors or angiotensin receptor blockers (ARBs) (20). ACE inhibitors and ARBs dilate the arteriole exiting the glomerulus, thus reducing blood pressure within the glomerular capillaries. ACE inhibitors and ARBs may attenuate the severity of DN, however, they do not stop the progression of the disease (21).

In mouse models for both Type I and T2D, a ketogenic diet has been shown to potentially reverse DN, when assessed by albumin:creatinine ratios and expression of stress-induced genes. Thus, there are mechanism(s) for kidney repair that suggest that DN is potentially reversible. As such, this evidence highlights why it is so important to frequently screen for kidney disease progression. Thus, if clinicians could stratify which diabetic patients are likely to progress to more serious kidney disease e.g. DN (22), more aggressive patient management and less healthcare resources would be required. One such approach is to study biomarkers involved in the pathophysiology of kidney disease.

Subclinical inflammation and oxidative stress have been implicated in the pathogenesis of DN (23). Furthermore, patients with T2D normally present with elevated proinflammatory cytokines and chemokines (24, 25).

Fortunately, not all diabetic patients will develop kidney disease. However, identifying patients who are at risk is challenging and current biomarkers do not always precede declining renal function (26). To date, no biomarker, or combination of biomarkers have been identified that can clinically predict the progression of DN.

Recently we identified a panel of biomarkers that predicted renal damage in patients undergoing cardiac surgery and orthopaedic surgery (27, 28). Using a similar approach to our previous studies, this pilot study attempted to identify potentially diagnostic and predictive biomarkers to stratify risk of DN progression. A justification of serum and urine biomarkers chosen in this study are described in **Supplementary 1**.

## Materials and Methods

### Study Participants

Study participants (N=88; control n=26; T2D n=32; DN n=30) were recruited between 2019 and 2020 from Whiteabbey Hospital Diabetic Clinic, Belfast, UK; the renal unit at Antrim Area Hospital, Antrim, UK; Ulster University (UU), Belfast, UK; and the University of the Third Age (U3A), Belfast, UK. Study participants were asked to complete a questionnaire to obtain information on their medical history and lifestyle/behaviours (smoking habits and alcohol consumption). Anthropometric details were also recorded for each patient to include height (cm), weight (kg) and blood pressure (mmHg). Blood pressure measurements were taken immediately following completion of the recruitment questionnaire. Estimated glomerular filtration rate and HbA1c were also recorded. Exclusion criteria included participants <18 years of age with autoimmune disease or who had been diagnosed with a condition or illness that could impact kidney function e.g., hepatitis B or C, polycystic kidney disease and/or stones. The study was approved by Ulster University School of Biomedical Sciences Filter Committee and University Research Governance and the Northern Health and Social Care Trust. Formal written informed consent was obtained from all participants and samples were collected in an outpatient setting. Participants received detailed information on the proposed study and were also invited to ask questions. The study was conducted according to the Standards for Reporting Diagnostic Accuracy (STARD) guidelines to facilitate interstudy comparison (29).

### Participant Sample Collection

Venous blood (10 ml) and urine (10 ml) (where possible) was collected from all DN patients. Venous blood (20 ml) and urine (10 ml) samples were obtained from all other study participants. Blood samples were stored at room temperature for 30 to 60 minutes to allow clot formation. Vacutainers were centrifuged at 1300 g for 10 minutes at 4°C and the serum was carefully decanted and aliquoted into prelabelled cryovials and stored at -80°C until analysis. The urine was aliquoted into prelabelled cryovials and stored at -80°C until time of analysis.

### Point of Care Assay

Aution sticks (10AE) were used for dipstick urinalysis and were interpreted using a PocketChem analyzer from Arkray Inc., Japan, according to manufacturer’s instructions.

### Bradford Assay (Urinary Protein)

Total urinary protein levels (mg/ml) were determined, in duplicate, by Bradford assay (Pierce, Rockford, IL, USA) using a stock solution of BSA (Sigma) as standard (2 mg/ml). Patient urine samples (10 μl/patient), after centrifugation (1200 g, 10 minutes, 4°C), were mixed with Bradford reagent (1 ml) and allowed to stand for 5 minutes. The samples were read at room temperature on a Hitachi Spectrophotometer (Model No. U-2800) at a wavelength of A_595_nm. Total urinary protein (mg/ml) was determined from a BSA calibration chart (1 to 5 mg/ml).

### Osmolality

Urine osmolality (mOsm) was determined, in duplicate, for each study participant using a Lӧser Micro-osmometer according to manufacturer’s instructions (Loser Messtechnik, Berlin, Germany).

### Biomarker Measurements

#### Biochip Array Technology

Biochip Array Technology (BAT) was used by Randox Clinical Laboratory Services (RCLS), Randox Science Park, Antrim, Northern Ireland, UK for the simultaneous detection of multiple biomarkers from a serum and/or urine patient sample (30) for the following proteins (abbreviations and limit of assay detection are shown in parenthesis): liver-type fatty acid-binding protein-1 (L-FABP) (1.06 ng/ml), macrophage inflammatory protein-1 alpha (MIP-1α) (0.68 pg/ml), soluble tumour necrosis factor receptor 1 (sTNFR1) (0.05 ng/ml) and sTNFR2 (0.01 ng/ml) were analysed on a Chronic Kidney Disease 1 (CKD1) biochip array. C-reactive protein (CRP) (500 ng/ml), C3a des Arg (C3DA) (30 ng/ml), neutrophil gelatine-associated lipocalin (NGAL) (3.52 ng/ml) and adiponectin (ADP) (39.06 ng/ml) were analysed on a Chronic Kidney Disease 2 (CKD2) biochip array. Interleukin (IL)-2 (2.97 pg/ml), -4 (2.12 pg/ml), -6 (0.12 pg/ml), -8 (0.36 pg/ml), -10 (0.37 pg/ml), -1α (0.19 pg/ml), -1β (0.26 pg/ml), vascular endothelial growth factor (VEGF) (3.24 pg/ml), interferon-γ (IFNγ) (0.44 pg/ml), monocyte chemoattractant protein-1 (MCP-1) (3.53 pg/ml), epithelial growth factor (EGF) (1.04 pg/ml) and tumour necrosis factor α (TNFα) (0.59 pg/ml) were measured on a high-sensitivity cytokine (hs-CTK) biochip array. Clusterin (10.82 ng/ml), cystatin C (0.87 ng/ml), NGAL (0.4 ng/ml) and kidney injury molecule 1 (KIM-1) (54.6 pg/ml) were analysed in urine only using the Acute Kidney Injury (AKI) biochip array.

### Other Biomarker Assays

The following biomarkers were measured in serum; transferrin (0.08 g/l), microalbumin (5.11 mg/l), heart-type fatty acid-binding protein (H-FABP) (2.94 ng/ml), cystatin C (0.4 mg/l), albumin (3.2 g/l), total antioxidant status (TAS) (0.21 mmol/l), urea (0.51 mmol/l) and creatinine (11.4 μmol/l) using the Imola analyzer (Randox Laboratories Ltd, Crumlin, UK), according to the manufacturer’s instructions. HDL cholesterol (HDL) (0.189 mmol/l), LDL cholesterol (LDL) (0.189 mmol/l), total cholesterol (0.865 mmol/l), triglycerides (22.9 mg/dl) and creatinine (311 μmol/l) were measured on a Daytona analyzer (Randox Laboratories Ltd, Crumlin, UK). Insulin (2.78 pmol/l) was measured using a Cobas e801 analyzer (Roche, Basel, Switzerland). Microalbumin and creatinine were also measured in urine.

### Midkine ELISA

Urine and serum midkine concentrations were measured using a commercial ELISA from LyraMid, Sydney, Australia, according to the manufacturer’s instructions.

### Statistical Analysis

Statistical analysis was undertaken using R (31). Data below the limit of detection (LOD) or mean detectable dose (MDD) was inputted as 90% of the LOD or MDD (28). Biomarker data were analysed using Kruskal-Wallis to identify which factors were differentially expressed between control, T2D and DN groups. Statistical significance was taken at the p<0.05 level and results are presented as mean ± SD where appropriate. Spearman’s rho correlations were also performed. Correlations ≥ 0.7 were considered significant. Area under the receiver operator characteristic (AUROC) was also calculated for each biomarker comparing control to T2D and T2D to DN patients.

## Results

### Participant Information

Participant demographics, anthropometric measurements, clinical information, medications, and participant behaviours are described in **Table 1**.

**Table 1:** Clinical and demographic characteristics of the study participants.

Factor	Control	Type 2 Diabetic	Diabetic Nephropathy	p value
	Mean ± SD	Mean ± SD	Mean ± SD	
Age	59.0 ± 13.8 (n=26)	62.2 ± 9.1 (n=32)	73.1 ± 9.0 (n=30)	<0.001
Alcohol (No)	3/26 (11.5%)	4/32 (12.5%)	8/30 (26.7%)	0.224
Bladder Repair (Yes)	0/26 (0%)	0/32 (0%)	1/30 (3.3%)	0.376
BMI	26.3 ± 3.9 (n=25)	34.4 ± 6.8 (n=16)	33.8 ± 6.1 (n=10)	<0.001
BP Diastolic (mmHg)	78.2 ± 10.1 (n=24)	81.3 ± 8.4 (n=32)	75.3 ± 11.2 (n=30)	0.065
BP Systolic (mmHg)	125.4 ± 13.8 (n=24)	139.1 ± 19.4 (n=32)	146.9 ± 23.9 (n=30)	0.001
Cancer (No)	25/26 (96.2%)	31/32 (96.9%)	29/30 (96.7%)	0.558
Cardiovascular Family History (1 or more)	11/26 (42.3%)	14/32 (43.8%)	12/29 (41.4%)	0.982
Cardiovascular Comorbidities (Yes (includes suspected))	4/26 (15.4%)	9/32 (28.1%)	18/30 (60%)	0.001
Diabetes DN Family History (1 or more)	0/26 (0%)	1/32 (3.1%)	4/29 (13.8%)	0.065
Diabetes Duration		10.8 ± 5.8 (n=32)	15.0 ± 6.3 (n=13)	0.063
Diabetes Family History (1 or more)	7/26 (26.9%)	19/32 (59.4%)	16/29 (55.2%)	0.032
Distal renal tubular acidosis (Yes)	0/26 (0%)	0/32 (0%)	1/30 (3.3%)	0.376
Enlarged Prostate (Yes (includes suspected))	0/6 (0%)	5/20 (25%)	7/18 (38.9%)	0.171
Hypertensive Medication (Yes)	6/26 (23.1%)	26/32 (81.2%)	27/30 (90%)	<0.001
Kidney Cysts (Yes)	1/26 (3.8%)	0/32 (0%)	2/30 (6.7%)	0.348
Kidney Infection (Yes (ever) (includes suspected))	8/26 (30.8%)	5/32 (15.6%)	7/30 (23.3%)	0.390
Kidney stones (Yes (ever) (includes suspected))	0/26 (0%)	4/32 (12.5%)	6/30 (20%)	0.061
Neuropathy (Yes (includes suspected))	0/26 (0%)	5/32 (15.6%)	5/30 (16.7%)	0.093
Retinopathy (Yes (includes suspected))	0/26 (0%)	6/32 (18.8%)	14/30 (46.7%)	<0.001
Sex (Male)	6/26 (23.1%)	20/32 (62.5%)	18/30 (60%)	0.005
Smoker (Current)	0/26 (0%)	5/32 (15.6%)	5/30 (16.7%)	0.003
Smoker (No)	19/26 (73.1%)	16/32 (50%)	7/30 (23.3%)	0.003
Smoker (Past)	7/26 (26.9%)	11/32 (34.4%)	18/30 (60%)	0.003
Statin Medication (Yes)	5/26 (19.2%)	23/32 (71.9%)	23/30 (76.7%)	<0.001
Weight (kg)	73.0 ± 11.5 (n=25)	99.3 ± 25.7 (n=30)	89.1 ± 21.4 (n=22)	<0.001

There were significantly more males in the T2D and DN groups; DN subjects were significantly older, T2D and DN subjects had significantly higher systolic blood pressure; and there were more smokers in the T2D and DN groups. As such, there were more T2D and DN patients prescribed medications for hypertension and cholesterol reduction. In addition, DN participants had the highest incidence of retinopathy, cardiovascular disease, prostatic hyperplasia, and kidney stones.

### Dip Stick Urinalysis Results

Urinary glucose, pH and protein levels were significantly different across groups (Kruskal-Wallis) (**Supplementary 2**).

### Biomarker Results

Identifying T2D patients at risk of progression to more serious kidney disease (e.g., ESRD) is clinically important so that aggressive treatments can be introduced to prevent irreversible damage to the kidney. This pilot study investigated both serum- and urine-based biomarkers to identify a potential biomarker or biomarker combination(s) with practicable clinical utility. Progression of kidney disease, in this pilot study, was based on declining eGFR function and increasing serum creatinine levels.

### Serum Biomarkers

In total, 34 serum biomarkers were investigated; 25/34 (73.5%) were significantly different across patient groups (Kruskal-Wallis) (**Table 2**) and across gender (**Supplementary 3**).

**Table 2:** Serum biomarkers (mean ± SD).

Factor	Control	Type 2 Diabetic	Diabetic Nephropathy	p value
	Mean ± SD	Mean ± SD	Mean ± SD	
ACR	0.7 ± 0.1 (n=26)	0.7 ± 0.2 (n=31)	0.3 ± 0.2 (n=28)	<0.001
Adiponectin (ng/ml)	8310.6 ± 4035.8 (n=18)	3650.7 ± 2114.8 (n=21)	6377.3 ± 4764.5 (n=20)	<0.001
Albumin (g/L)	43.5 ± 1.9 (n=26)	44.6 ± 2.0 (n=32)	41.2 ± 6.2 (n=28)	<0.001
Creatinine (μmol/L)	63.3 ± 11.3 (n=26)	64.6 ± 15.3 (n=31)	185.8 ± 87.1 (n=28)	<0.001
CRP (ng/ml)	2454.8 ± 5046.7 (n=18)	4779.7 ± 4583.5 (n=21)	9563.9 ± 7412.9 (n=20)	<0.001
Cystatin C (mg/L)	0.8 ± 0.2 (n=26)	0.9 ± 0.2 (n=32)	1.9 ± 0.6 (n=28)	<0.001
EGF (pg/ml)	71.2 ± 47.1 (n=26)	36.5 ± 36.0 (n=31)	21.1 ± 32.9 (n=28)	<0.001
eGFR	93.3 ± 18.6 (n=26)	105.1 ± 30.0 (n=31)	36.5 ± 22.4 (n=28)	<0.001
H-FABP (ng/ml)	4.5 ± 1.9 (n=26)	5.6 ± 2.1 (n=30)	17.9 ± 7.7 (n=28)	<0.001
L-FABP (ng/ml)	1.1 ± 1.8 (n=26)	2.9 ± 4.1 (n=32)	7.6 ± 5.8 (n=28)	<0.001
Glucose (mmol/L)		11.2 ± 3.6 (n=30)	12.4 ± 3.9 (n=12)	0.242
HbA1c (mmol/mol)		70.9 ± 12.1 (n=29)	72.8 ± 18.9 (n=20)	0.984
HDL Cholesterol (mmol/L)	1.7 ± 0.4 (n=26)	1.2 ± 0.3 (n=32)	1.2 ± 0.4 (n=28)	<0.001
IFN-γ (pg/ml)	3.1 ± 14.3 (n=26)	0.4 ± 0.5 (n=31)	0.5 ± 0.7 (n=28)	0.822
IL-10 (pg/ml)	0.6 ± 0.4 (n=26)	0.6 ± 0.3 (n=31)	0.9 ± 0.6 (n=28)	0.001
IL-1α (pg/ml)	0.2 ± 0.2 (n=26)	0.1 ± 0.1 (n=31)	0.2 ± 0.3 (n=28)	0.753
IL-1β (pg/ml)	1.2 ± 1.0 (n=26)	1.0 ± 0.5 (n=31)	1.3 ± 1.6 (n=28)	0.668
IL-2 (pg/ml)	1.2 ± 1.6 (n=26)	0.6 ± 0.7 (n=31)	0.7 ± 0.8 (n=28)	0.557
IL-4 (pg/ml)	1.8 ± 1.4 (n=26)	1.3 ± 0.4 (n=31)	1.8 ± 3.1 (n=28)	<0.001
IL-6 (pg/ml)	1.1 ± 0.8 (n=26)	3.4 ± 6.8 (n=31)	7.5 ± 11.9 (n=28)	<0.001
IL-8 (pg/ml)	6.4 ± 3.3 (n=26)	11.3 ± 8.8 (n=31)	21.9 ± 53.7 (n=28)	0.005
Insulin (pmol/L)	143.9 ± 208.7 (n=26)	147.9 ± 103.5 (n=32)	184.2 ± 156.8 (n=28)	0.055
LDL Cholesterol (mmol/L)	2.7 ± 0.8 (n=26)	2.6 ± 0.7 (n=32)	2.0 ± 1.0 (n=28)	0.008
MCP-1 (pg/ml)	172.3 ± 73.8 (n=26)	216.7 ± 86.5 (n=31)	211.1 ± 91.0 (n=28)	0.178
Midkine (pg/ml)	90.0 ± 132.5 (n=26)	176.8 ± 422.5 (n=32)	2426.9 ± 3479.4 (n=28)	<0.001
MIP-1α (pg/ml)	5.2 ± 1.9 (n=26)	8.6 ± 6.0 (n=32)	10.8 ± 3.9 (n=28)	<0.001
NGAL (ng/ml)	56.9 ± 18.0 (n=18)	49.0 ± 15.7 (n=21)	138.8 ± 85.5 (n=20)	<0.001
sTNFR1 (ng/ml)	0.5 ± 0.2 (n=26)	0.7 ± 0.2 (n=32)	2.7 ± 1.7 (n=28)	<0.001
sTNFR2 (ng/ml)	1.1 ± 0.4 (n=26)	1.7 ± 0.7 (n=32)	6.5 ± 3.5 (n=28)	<0.001
TAS (mmol/L)	1.7 ± 0.1 (n=26)	1.8 ± 0.1 (n=32)	1.8 ± 0.3 (n=28)	0.004
TNFα (pg/ml)	3.5 ± 5.6 (n=26)	2.9 ± 1.0 (n=31)	4.8 ± 2.5 (n=28)	<0.001
Total Cholesterol (mmol/L)	5.1 ± 0.8 (n=26)	4.5 ± 1.0 (n=32)	3.9 ± 1.1 (n=28)	<0.001
Transferrin (g/L)	2.4 ± 0.3 (n=26)	2.8 ± 0.3 (n=32)	2.2 ± 0.7 (n=28)	<0.001
Triglyceride (mmol/L)	1.2 ± 0.5 (n=26)	2.5 ± 2.1 (n=32)	1.8 ± 0.8 (n=28)	<0.001
Urea (mmol/L)	5.5 ± 1.4 (n=26)	5.6 ± 1.3 (n=32)	8.0 ± 4.6 (n=28)	0.025
VEGF (pg/ml)	89.2 ± 71.1 (n=26)	77.2 ± 53.0 (n=31)	103.4 ± 113.7 (n=28)	0.903

### Urine Biomarkers

In total, 25 urine biomarkers were investigated; 13/25 (52.0%) were significantly different across patient groups (Kruskal-Wallis) (**Table 3**) and across gender (**Supplementary 3**).

**Table 3:** Urine biomarkers (mean ± SD).

Factor	Control	Type 2 Diabetic	Diabetic Nephropathy	p value
	Mean ± SD	Mean ± SD	Mean ± SD	
ACR	4.3 ± 7.5 (n=26)	2.7 ± 4.2 (n=29)	19.7 ± 16.8 (n=10)	0.001
Clusterin (ng/ml)	164.2 ± 206.5 (n=26)	93.5 ± 162.5 (n=29)	165.4 ± 240.5 (n=10)	0.190
Creatinine (μmol/L)	6835.3 ± 5860.7 (n=26)	6463.2 ± 2721.1 (n=29)	6539.5 ± 3258.3 (n=10)	0.746
Cystatin C (ng/ml)	15.3 ± 20.9 (n=26)	16.6 ± 11.9 (n=29)	32.0 ± 26.5 (n=10)	0.027
EGF (pg/ml)	595.4 ± 404.8 (n=26)	516.0 ± 0.0 (n=29)	653.6 ± 435.3 (n=10)	0.290
L-FABP (ng/ml)	35.7 ± 14.7 (n=26)	11.3 ± 16.0 (n=29)	29.3 ± 24.7 (n=10)	<0.001
IFN-γ (pg/ml)	0.0 ± 0.1 (n=26)	0.0 ± 0.1 (n=29)	0.1 ± 0.2 (n=10)	0.985
IL-10 (pg/ml)	0.4 ± 0.2 (n=26)	0.5 ± 0.1 (n=29)	0.5 ± 0.1 (n=10)	0.020
IL-1α (pg/ml)	6.7 ± 12.6 (n=26)	1.1 ± 1.6 (n=29)	2.2 ± 4.1 (n=10)	0.002
IL-1β (pg/ml)	13.3 ± 47.5 (n=26)	1.7 ± 1.8 (n=29)	11.7 ± 24.7 (n=10)	0.042
IL-2 (pg/ml)	0.5 ± 0.4 (n=26)	0.5 ± 0.5 (n=29)	0.9 ± 0.5 (n=10)	0.072
IL-4 (pg/ml)	1.9 ± 0.3 (n=26)	1.8 ± 0.2 (n=29)	1.7 ± 0.2 (n=10)	0.088
IL-6 (pg/ml)	9.2 ± 24.6 (n=26)	3.1 ± 5.9 (n=29)	3.0 ± 3.0 (n=10)	0.264
IL-8 (pg/ml)	273.0 ± 769.2 (n=26)	42.7 ± 76.7 (n=29)	279.4 ± 683.5 (n=10)	0.024
KIM-1 (pg/ml)	1425.9 ± 3090.5 (n=26)	1464.7 ± 1598.4 (n=29)	2961.4 ± 3694.3 (n=10)	0.087
MCP-1 (pg/ml)	112.6 ± 161.6 (n=26)	97.9 ± 82.9 (n=29)	77.3 ± 30.7 (n=10)	0.472
Microalbumin (mg/L)	18.9 ± 38.8 (n=26)	14.7 ± 19.0 (n=29)	119.9 ± 98.0 (n=10)	0.006
Midkine (pg/ml)	111.9 ± 238.0 (n=26)	1525.7 ± 2568.7 (n=29)	1443.3 ± 1105.9 (n=10)	<0.001
MIP-1α (pg/ml)	19.5 ± 7.1 (n=26)	5.7 ± 7.7 (n=29)	11.0 ± 9.5 (n=10)	<0.001
NGAL (ng/ml)	83.0 ± 185.9 (n=26)	36.8 ± 46.2 (n=29)	71.1 ± 70.8 (n=10)	0.123
Osmolality (mOsm)	460.8 ± 239.6 (n=26)	811.9 ± 207.1 (n=29)	557.6 ± 187.7 (n=10)	<0.001
Protein (mg/ml)	0.1 ± 0.1 (n=26)	0.1 ± 0.0 (n=29)	0.3 ± 0.2 (n=10)	0.005
sTNFR1 (ng/ml)	0.9 ± 0.6 (n=26)	1.5 ± 0.7 (n=29)	2.7 ± 2.0 (n=10)	<0.001
sTNFR2 (ng/ml)	2.2 ± 2.3 (n=26)	4.9 ± 2.6 (n=29)	7.4 ± 3.9 (n=10)	<0.001
TNFα (pg/ml)	3.5 ± 0.9 (n=26)	3.3 ± 0.7 (n=29)	3.1 ± 0.5 (n=10)	0.543
VEGF (pg/ml)	69.4 ± 75.3 (n=26)	51.7 ± 59.7 (n=29)	89.2 ± 53.7 (n=10)	0.051

### Biomarkers That Differentiated Control From T2D, and T2D From DN Subjects

**Figure 1:**
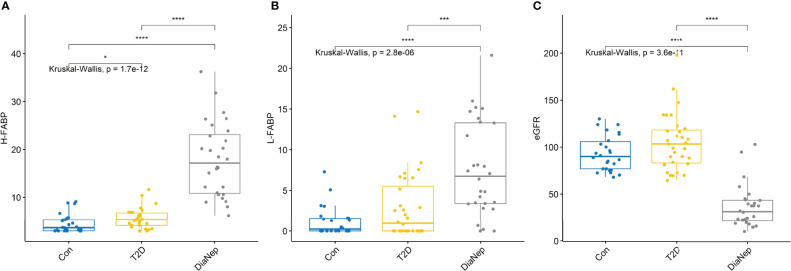
**(A)** Serum H-FABP was significantly higher in the T2D and DN patients with respect to control participants. **(B)** Serum L-FABP was also significantly higher in the DN patients with respect to control participants. However, although the L-FABP was significantly different between the T2D and DN patients, there was no difference in L-FABP between the control and T2D participants. **(C)** eGFR for control and T2D participants were not significantly different. However, there was almost a 70% decrease in eGFR in the DN patients with respect to control and T2D participants. Stars of significance *p<0.05, ***p<0.001, and ****p<0.0001.

The AUROC for each of the biomarkers that differentiated control from T2D, and T2D from DN subjects, are described in **Table 4**. In this study, we aimed to investigate biomarkers which were differentially expressed in patients with T2D and DN. Biomarkers with an AUROC ≥0.84 were considered significant, in this study. Biomarkers which met this criterion included creatinine, cystatin C, H-FABP, midkine, NGAL, sTNFR1 and sTNFR2. Serum H-FABP and L-FABP increased across groups with the most significant increase observed between T2D and DN patients (**Figures 1A, B**). Unsurprisingly, eGFR levels were not significantly different between control and T2D however, when we compared T2D with DN, the eGFR levels were decreased almost 70% (**Figure 1C**).

**Table 4:** AUROC for control vs. T2D and T2D vs. DN.

Serum Biomarker	AUROC (95% CI)	
	Control vs. Type 2 Diabetic	Type 2 Diabetic vs. Diabetic Nephropathy
ACR	0.517 (0.362-0.673)	0.947 (0.879-1.000)
Adiponectin	0.881 (0.772-0.990)	0.695 (0.528-0.862)
Albumin	0.677 (0.535-0.820)	0.788 (0.658-0.918)
Creatinine	0.531 (0.376-0.686)	0.954 (0.894-1.000)
CRP	0.749 (0.586-0.911)	0.702 (0.540-0.865)
Cystatin C	0.708 (0.571-0.845)	0.966 (0.914-1.000)
EGF	0.730 (0.598-0.863)	0.711 (0.574-0.849)
H-FABP	0.694 (0.551-0.837)	0.974 (0.941-1.000)
L-FABP	0.581 (0.439-0.722)	0.775 (0.656-0.893)
HDL Cholesterol	0.872 (0.782-0.962)	0.563 (0.409-0.716)
IFN-γ	0.500 (0.343-0.657)	0.544 (0.394-0.694)
IL-10	0.594 (0.442-0.745)	0.695 (0.560-0.831)
IL-1α	0.530 (0.375-0.685)	0.534 (0.383-0.685)
IL-1β	0.535 (0.375-0.695)	0.547 (0.395-0.698)
IL-2	0.580 (0.430-0.730)	0.532 (0.385-0.679)
IL-4	0.760 (0.627-0.893)	0.554 (0.403-0.705)
IL-6	0.775 (0.652-0.898)	0.776 (0.655-0.898)
IL-8	0.745 (0.618-0.873)	0.503 (0.350-0.657)
Insulin	0.654 (0.504-0.804)	0.463 (0.311-0.615)
LDL Cholesterol	0.578 (0.425-0.730)	0.679 (0.539-0.820)
MCP-1	0.638 (0.492-0.784)	0.549 (0.397-0.701)
Midkine	0.750 (0.611-0.889)	0.905 (0.819-0.990)
MIP-1α	0.791 (0.670-0.911)	0.705 (0.572-0.838)
NGAL	0.646 (0.467-0.824)	0.890 (0.766-1.000)
sTNFR1	0.757 (0.630-0.883)	0.948 (0.877-1.000)
sTNFR2	0.770 (0.650-0.889)	0.943 (0.884-1.000)
TAS	0.792 (0.668-0.916)	0.545 (0.386-0.703)
TNFα	0.633 (0.484-0.782)	0.825 (0.717-0.933)
Total Cholesterol	0.719 (0.582-0.855)	0.653 (0.510-0.797)
Transferrin	0.773 (0.651-0.894)	0.797 (0.670-0.925)
Triglyceride	0.805 (0.694-0.917)	0.592 (0.446-0.738)
Urea	0.531 (0.377-0.685)	0.672 (0.523-0.821)
VEGF	0.465 (0.306-0.623)	0.487 (0.328-0.645)

**Figure 2:**
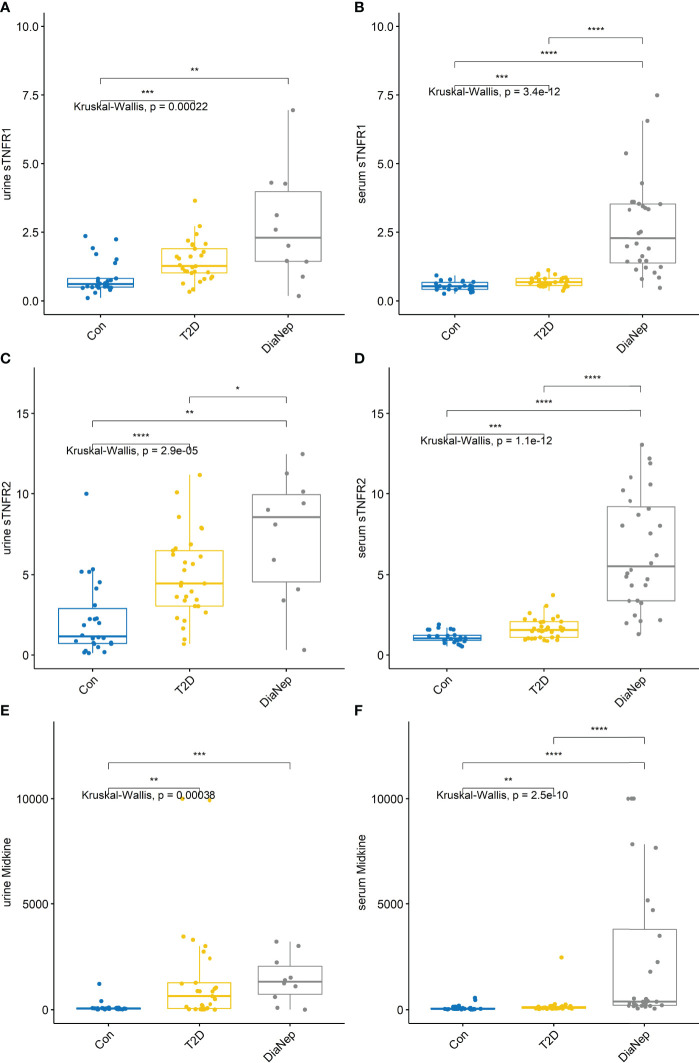
Urine and serum levels for sTNFR1 and 2 and midkine increased across all groups **(A)** urine sTNFR1, **(B)** serum sTNFR1, **(C)** urine sTNFR2, **(D)** serum sTNFR2, **(E)** urine midkine, **(F)** serum midkine. Stars of significance *p<0.05, **p<0.01, ***p<0.001, and ****p<0.0001.

Urine and serum sTNFR1, sTNFR2 and midkine exhibited the same pattern observed for H-FABP (**Figure 2**). Urine midkine levels were significantly different between control and the other groups. However, urine midkine levels in T2D were not significantly different from DN patients. However, serum midkine levels were significantly higher in the DN patients when compared to T2D, suggesting that systemic levels of midkine are increasing because of kidney dysfunction, as noted by the decline in eGFR.

### Correlations

**Figure 3:**
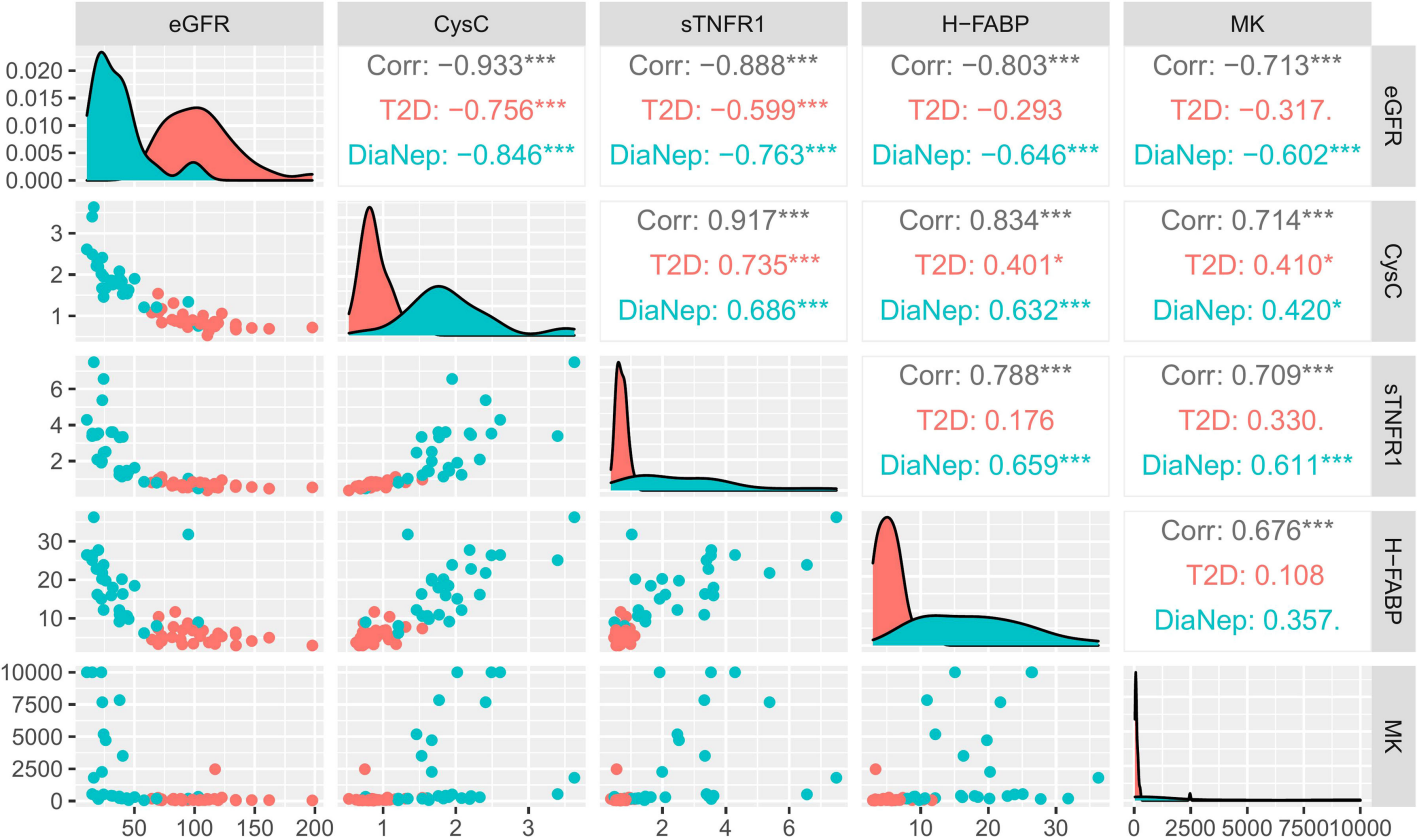
Correlation matrix chart for significant serum biomarkers (T2D vs. DN) and eGFR. Scatterplots of each pair of numeric variable are drawn on the left part of the figure. Spearman’s rho correlations are displayed on the right. Variable distributions are shown on the diagonal. The results are coloured by cohort, i.e., T2D (red), DN (green) and both groups combined (grey). Correlations ≥ 0.7 were considered significant. Stars of significance *p<0.05, ***p<0.001.

Serum biomarker correlations with eGFR for T2D vs. DN participants, demonstrated that only serum biomarkers, as expected, correlated with eGFR (**Figure 3**) (Spearman’s rho).

## Discussion

Diabetic nephropathy is a serious progressive pathology that requires immediate medical intervention. However, identifying diabetic patients that will progress to DN remains challenging as the underlying pathophysiology of the disease is unclear. Furthermore, albuminuria and decreased kidney function (eGFR), standard end points that are employed clinically to diagnose DN, only identify the disease once established. Currently, there are no biomarkers that can predict progression in T2D to DN. Biomarkers that could help identify which patients are at risk of progression would allow clinicians to implement early and aggressive treatment management. Furthermore, biomarkers that are predictive would also allow monitoring of therapeutic intervention and effectiveness of treatment regimens.

In this pilot study, we compared the expression of 34 biomarkers in serum and 25 in urine, known to be involved in renal disease (**Supplementary 1**). Twenty-five serum and 13 urine biomarkers were differentially expressed across patient groups. To determine the extent of kidney dysfunction, eGFR was measured and compared in control, T2D and DN participants. eGFR for control and T2D participants were not significantly different. However, there was almost a 70% decrease in eGFR in the DN subjects with respect to control and T2D participants (**Figure 1C**). The following serum biomarkers were inversely correlated with eGFR; midkine, sTNFR1, sTNFR2, H-FABP and cystatin C (**Figure 3**). None of the urine biomarkers investigated correlated with eGFR (data not shown).

Midkine is a heparin-binding growth factor of low molecular weight that forms a family with pleiotrophin (NEGF1, 46% homologous with midkine). Midkine is a non-glycosylated protein, composed of two domains held by disulphide bridges. Midkine is a developmentally important retinoic acid-responsive gene product strongly induced during *mid*-gestation, hence the name midkine. Midkine is restricted mainly to certain tissues in the normal adult. However, it is strongly induced during oncogenesis, inflammation, and tissue repair (32, 33).

The pathophysiological roles involving midkine are diverse, ranging from AKI to progression of CKD, often accompanied by hypertension, renal ischemia and DN (27, 28, 34). After ischemic reperfusion *in vivo*, midkine is immediately induced in the proximal tubules, leading to the up-regulation of macrophage inflammatory protein-2 (MIP-2) for neutrophils and monocyte chemotactic protein (MCP-1) for macrophages (35). Eventually, infiltrated inflammatory cells cause severe tubulointerstitial injury (35). Previous studies have shown that silencing renal midkine expression with anti-sense oligos prevents kidney damage following ischemia/reperfusion injury (36).

Urinary midkine levels were elevated however, they were not significantly different in T2D and DN patients with respect to control participants. Moreover, eGFR function was normal in both control and T2Ds. When we compared T2D with DN patients, eGFR function was compromised in the DN patients and the serum MK levels were significantly elevated with respect to both control and T2D participants. Thus, in T2Ds, the kidney function is still preserved and the eGFR is normal. However, progression to DN compromises the kidney, eGFR function decreases and systemic levels of serum MK increase. Therefore, the data would suggest that serum midkine could be a potential acute phase biomarker for T2D patients progressing to DN. Changes in systemic midkine levels i.e., increased circulating midkine, could be a prognostic indicator of potentially worsening kidney disease.

Serum and urine sTNFR1 and 2 levels increase in relation to deterioration in renal function (37). In individuals with T2D, increased sTNFR1 and 2 serum levels have been correlated with renal structural changes e.g. mesangial volume, interstitial volume, and glomerular sclerosis (38, 39). TNF-α is involved in cellular signalling, including apoptosis, by interacting with membrane receptors; sTNFR1 and 2. Activation of TNF pathways are associated with kidney disease progression in subjects with renal insufficiency (40). Therefore, it was unsurprising that both sTNFR1 and 2 levels were significantly higher in both T2D and DN subjects with respect to the control group. Furthermore, a doubling effect was noted for sTNFR1 and 2 between the T2Ds and DN subjects, indicating that more structural damage to the kidney was evident in the DN group.

Fatty acid-binding proteins are small cytoplasmic proteins (15kDa) that have been shown to be promising new biomarkers for detection of renal injury (41). H-FABP is found in distal tubular cells and L-FABP in proximal tubular cells (42). Evidence has shown that altered lipid metabolism, such as hyperlipidaemia and increased free fatty acids (FFAs) are an important characteristic of obesity that contributes to renal lesions (43). H-FABP is located mostly along the capillary wall in human glomeruli (44). Several studies have focused on the role of H-FABP in cardiac disease however, a recent study attempted to clarify H-FABP’s role in kidney disease (45). Overexpression of H-FABP in a podocyte cell line was associated with derangement of lipid metabolism, inflammation, and oxidative stress in podocytes (45). Inhibiting expression of H-FABP attenuated the damage suggesting that H-FABP could be a potential drug target to reduce glomerular injury in DN patients (45). However, elevated serum H-FABP in the DN subjects may also be related to cardiovascular disease, as almost 60% of these patients had a history of cardiovascular comorbidities.

Elevated serum L-FABP has been shown to positively correlate with obesity and insulin resistance (46). Therefore, it was unsurprising that T2D and DN patients had significantly higher triglycerides and an increased BMI.

Cystatin C is a 13.3 kDa protein encoded by the *CST3* gene. Cystatin C is a biomarker of kidney function and for predicting new-onset or deteriorating cardiovascular disease (47). Cystatin C inversely correlates with eGFR. Therefore, elevated levels of circulating cystatin C are an early indicator of kidney dysfunction. In this study, no difference in circulating levels of cystatin C were observed between the control and T2D subjects. However, levels of cystatin C were two-fold higher in the DN subjects (48).

Unsurprisingly, pro-inflammatory cytokines IL-6, IL-8, IL-10, and CRP were elevated in the serum of both T2D and DN patients. However, they did not correlate with decreasing eGFR. Furthermore, none of these individual cytokine AUROC for differentiating between control and T2D and T2D and DN were ≥0.78.

## Conclusion

In conclusion, 5 biomarkers were identified: midkine, sTNFR1, sTNFR2, H-FABP and cystatin C to have clinical utility for differentiating patients with T2D from DN. A combination of these 5 biomarkers could have potential for use in the diabetic outpatient clinic to identify individuals at risk of kidney disease progression, allowing earlier, and more effective management intervention. Our results would need to be confirmed in longitudinal studies involving T2D’s and their potential progression to DN.

## Clinical Utility of the Biomarkers

Commonly employed methods to test if a biomarker will add to risk prediction models are normally based on (a) model discrimination, (b) model calibration and, (c) risk reclassification (49). Therefore, addition of the biomarkers sTNFR1 (or 2), H-FABP and midkine, to known risks for DN, would allow clinicians to both identify patients at risk of progression and monitor therapeutic intervention.

## Limitations of the Pilot Study

The main limitations of the study include (1) the small sample number of participants in each group, which in turn limited evaluating the biomarkers by gender, (2) the number of DN urine samples that were available for analysis, (3) and no staging information for DN patients. However, despite the limitations of this pilot study, the results warrant further investigation. Novel biomarkers that could be used for stratifying risk of progression of T2D to DN offer significant clinical utility and decreased morbidity and mortality for the patient. Furthermore, identifying patients at risk of progression allow better use of hospital resources.

## Data Availability Statement

The raw data supporting the conclusions of this article will be made available by the authors, without undue reservation.

## Ethics Statement

The studies involving human participants were reviewed and approved by Ulster University School of Biomedical Sciences Filter Committee and University Research Governance and the Northern Health and Social Care Trust. The patients/participants provided their written informed consent to participate in this study.

## Author Contributions

Concept and study design – DC, SB, FJ, SBr, MJK, JVL, PF, TM, and MWR; Statistical analysis – CH, JW, and MWR; Manuscript preparation – JW, MJK, JVL, PF, and MWR; Sample collection and processing – CH, SB, FJ, AS, and MWR; Manuscript review – CH, DC, SBr, SB, FJ, AS, JW, MJK, JVL, PF, TM, and MWR. All authors contributed to the article and approved the submitted version.

## Funding

This study was funded by the Randox Laboratories Ltd – Ulster University Industrial Ph.D. Academy.

## Conflict of Interest

The authors declare that this study received funding from Randox Laboratories Ltd as part of the Randox Laboratories Ltd – Ulster University PhD Academy Studentship. Randox had the following involvement in the study: analysis of patient samples, statistical analysis, supervision of the project, preparation of the manuscript, and the decision to publish.

SBr, JW, MJK, JVL, and MWR are employees of Randox Laboratories Ltd. but hold no shares in the company. PF is the managing director and owner of Randox Laboratories Ltd. A patent has been filed to protect the biomarker combination disclosed in the manuscript.

The remaining authors declare that the research was conducted in the absence of any commercial or financial relationships that could be construed as a potential conflict of interest.

## Publisher’s Note

All claims expressed in this article are solely those of the authors and do not necessarily represent those of their affiliated organizations, or those of the publisher, the editors and the reviewers. Any product that may be evaluated in this article, or claim that may be made by its manufacturer, is not guaranteed or endorsed by the publisher.
