# Supplementary material for: Biomarkers for Detecting Kidney Dysfunction in Type-2 Diabetics and Diabetic Nephropathy Subjects: A Case-Control Study to Identify Potential Biomarkers of DN to Stratify Risk of Progression in T2D Patients
Source: Front Endocrinol (Lausanne). 2022 Jun 29;13:887237. doi: 10.3389/fendo.2022.887237 (PMC9276980; doi:10.3389/fendo.2022.887237)
Supplement: Supplementary file 1 [file DataSheet_1.pdf]

Supplement 1

Factor	Role of Biomarker in T2D and DN
Adiponectin	Serum adiponectin levels are positively associated with diabetic peripheral neuropathy (1)
CRP	C-reactive protein (CRP) is associated with progressive diabetic nephropathy in patients with type-2 diabetes (T2DN) (2)
Cystatin C	Serum cystatin C is a useful marker of early renal impairment in type 2 diabetic patients because it reflects both a decrease in GFR and elevated ACR (3)
EGF	EGF can serve as an early marker of diabetes nephropathy in children and adolescents (4)
H-FABP	FABP may be a clinical marker to screen for kidney dysfunction and identify patients who are likely to experience deterioration in renal function in the future (5)
L-FABP	FABP may be a clinical marker to screen for kidney dysfunction and identify patients who are likely to experience deterioration in renal function in the future (5)
IFN-ɣ	IFN-gamma is involved in nephropathy complication of type 2 diabetes (6)
IL-10	Abnormal IL-10 expression whether transient or prolonged, as well as interactions with other growth factors as a response to diverse stimuli is linked to the appearance and progression of a variety of kidney disorders.(7)
IL-1α	IL-1α released from renal tubular cells is the key inflammatory molecule responsible for the renal inflammation in DN.(8)
IL-1β	Inflammasome-driven release of interleukin(IL)-1β is a central element of many forms of sterile inflammation and has been evident to promote the onset and progression of diabetic kidney disease (9)
IL-2	IL-2 activities reverse inflammation and protects from type-2 diabetic nephropathy through promoting T-regulatory cells and type 2 immune response.(10)
IL-4	Increased in situ production of IL-4 in DN (11)
IL-6	Increased in situ production of IL-6 in DN (11)
IL-8	Serum IL-8 level can be used for an early diagnosis of DN. (12)
MCP-1	The cellular mechanisms linking MCP-1 to kidney injury. (13)
Midkine	Midkine plays a critical role in the tubulointerstitial inflammation associated with diabetic nephropathy through activation of the MCP-1 pathway.(14)
MIP-1α	Renal expression of cytokines and chemokines in diabetic nephropathy (15)
NGAL	NGAL can be used as a biomarker to diagnose DN even earlier to incipient nephropathy.(16)
sTNFR1	sTNFR1 is predictive of acute kidney injury preoperatively in cardiac surgery (17)
sTNFR2	sTNFR2 is predictive of acute kidney injury post cardiac surgery (17)
TNFα	TNFα is an acute phase biomarker in AKI (17)
Transferrin	Urine transferrin correlates with subclinical atherogenesis in patients with t2DM (18)
VEGF	VEGF is involved in the pathogenesis of diabetic kidney disease (19)

References

1. Sun Q, Yan B, Yang D, Guo J, Wang C, Zhang Q, Shi Y, Shi X, Tian G, Liang X. Serum Adiponectin Levels Are Positively Associated With Diabetic Peripheral Neuropathy in Chinese Patients With Type 2 Diabetes. *Front Endocrinol (Lausanne)* (2020) **11**: doi: 10.3389/FENDO.2020.567959

2. You YK, Huang XR, Chen HY, Lyu XF, Liu HF, Lan HY. C-Reactive Protein Promotes Diabetic Kidney Disease in db/db Mice via the CD32b-Smad3-mTOR signaling Pathway. *Sci Rep* (2016) **6**: doi: 10.1038/SREP26740

3. Jeon Y La, Kim MH, Lee WI, Kang SY. Cystatin C as an early marker of diabetic nephropathy in patients with type 2 diabetes. *Clin Lab* (2013) **59**:1221–1229. doi: 10.7754/CLIN.LAB.2013.120804

4. Ledeganck KJ, den Brinker M, Peeters E, Verschueren A, De Winter BY, France A, Dotremont H, Trouet D. The next generation: Urinary epidermal growth factor is associated with an early decline in kidney function in children and adolescents with type 1 diabetes mellitus. *Diabetes Res Clin Pract* (2021) **178**:108945. doi: 10.1016/J.DIABRES.2021.108945

5. Tsai IT, Wu CC, Hung WC, Lee TL, Hsuan CF, Wei CT, Lu YC, Yu TH, Chung FM, Lee YJ, et al. FABP1 and FABP2 as markers of diabetic nephropathy. *Int J Med Sci* (2020) **17**:2338. doi: 10.7150/IJMS.49078

6. Nosratabadi R, Arababadi MK, Hassanshahi G, Yaghini N, Pooladvand V, Shamsizadeh A, Zarandi ER, Hakimi H. Evaluation of IFN-gamma serum level in nephropatic type 2 diabetic patients. *Pakistan J Biol Sci PJBS* (2009) **12**:746–749. doi: 10.3923/PJBS.2009.746.749

7. Sinuani I, Beberashvili I, Averbukh Z, Sandbank J. Role of IL-10 in the progression of kidney disease. *World J Transplant* (2013) **3**:91–8. doi: 10.5500/wjt.v3.i4.91

8. Salti T, Khazim K, Haddad R, Campisi-Pinto S, Bar-Sela G, Cohen I. Glucose Induces IL-1α-Dependent Inflammation and Extracellular Matrix Proteins Expression and Deposition in Renal Tubular Epithelial Cells in Diabetic Kidney Disease. *Front Immunol* (2020) **11**:1270. doi: 10.3389/FIMMU.2020.01270/BIBTEX

9. Lei Y, Devarapu SK, Motrapu M, Cohen CD, Lindenmeyer MT, Moll S, Kumar S V., Anders HJ. Interleukin-1β inhibition for chronic kidney disease in obese mice with type 2 diabetes. *Front Immunol* (2019) **10**:1223. doi: 10.3389/FIMMU.2019.01223/FULL

10. Sabapathy V, Stremska ME, Mohammad S, Corey RL, Sharma PR, Sharma R. Novel immunomodulatory cytokine regulates inflammation, diabetes, and obesity to protect from diabetic nephropathy. *Front Pharmacol* (2019) **10**:572. doi: 10.3389/FPHAR.2019.00572/BIBTEX

11. Araújo LS, Torquato BGS, Da Silva CA, Dos Reis Monteiro MLG, Dos Santos Martins ALM, Da Silva MV, Dos Reis MA, MacHado JR. Renal expression of cytokines and chemokines in diabetic nephropathy. *BMC Nephrol* (2020) **21**:1–11. doi: 10.1186/S12882-020-01960-0/FIGURES/5

12. Liu S yan, Chen J, Li Y feng. Clinical significance of serum interleukin-8 and soluble tumor necrosis factor-like weak inducer of apoptosis levels in patients with diabetic nephropathy. *J Diabetes Investig* (2018) **9**:1182–1188. doi: 10.1111/JDI.12828

13. Giunti S, Barutta F, Cavallo Perin P, Gruden G. Targeting the MCP-1/CCR2 System in diabetic kidney disease. *Curr Vasc Pharmacol* (2010) **8**:849–860. doi:

10.2174/157016110793563816

14. Kosugi T, Yuzawa Y, Sato W, Arata-Kawai H, Suzuki N, Kato N, Matsuo S, Kadomatsu K. Midkine is involved in tubulointerstitial inflammation associated with diabetic nephropathy. *Lab Investig 2007 879* (2007) **87**:903–913. doi: 10.1038/labinvest.3700599

15. Araújo LS, Torquato BGS, Da Silva CA, Dos Reis Monteiro MLG, Dos Santos Martins ALM, Da Silva MV, Dos Reis MA, MacHado JR. Renal expression of cytokines and chemokines in diabetic nephropathy. *BMC Nephrol* (2020) **21**: doi: 10.1186/S12882-020-01960-0

16. Kaul A, Behera M, Rai M, Mishra P, Bhaduaria D, Yadav S, Agarwal V, Karoli R, Prasad N, Gupta A, et al. Neutrophil Gelatinase-associated Lipocalin: As a Predictor of Early Diabetic Nephropathy in Type 2 Diabetes Mellitus. *Indian J Nephrol* (2018) **28**:53. doi: 10.4103/IJN.IJN_96_17

17. McBride WT, Kurth MJ, McLean G, Domanska A, Lamont J V., Maguire D, Watt J, Fitzgerald P, Young I, Joseph J, et al. Stratifying risk of acute kidney injury in pre and post cardiac surgery patients using a novel biomarker-based algorithm and clinical risk score. *Sci Reports 2019 91* (2019) **9**:1–12. doi: 10.1038/s41598-019-53349-1

18. Sánchez-Hidalgo JJ, Suárez-Cuenca JA, Lozano-Nuevo JJ, García-López VH, Leal-Gutiérrez MG, León-Angel SA, Ramírez-Villa ML, Rodea-Rubio ME, González-Hernández JE, Canela-Mayoral JA, et al. Urine transferrin as an early endothelial dysfunction marker in type 2 diabetic patients without nephropathy: a case control study. *Diabetol Metab Syndr* (2021) **13**:1–8. doi: 10.1186/S13098-021-00745-1/TABLES/2

19. Khamaisi M, Schrijvers BF, De Vriese AS, Raz I, Flyvbjerg A. The emerging role of VEGF in diabetic kidney disease. *Nephrol Dial Transplant* (2003) **18**:1427–1430. doi: 10.1093/NDT/GFG242

Supplement 2

Participant Aution dipstick urinalysis results (POC assay)

	Control	Type 2 Diabetes (T2D)	Diabetic Nephropathy (DN)	
**Factor**	**Mean ± SD**	**Mean ± SD**	**Mean ± SD**	**p value**
Blood (≥0.6 mg/dL)	3/26 (11.5%)	0/29 (0%)	1/10 (10%)	0.177
Glucose (≥100 mg/dL)	0/26 (0%)	25/29 (86.2%)	4/10 (40%)	<0.001
Leukocytes (≥75 Leu/µl)	7/26 (26.9%)	2/29 (6.9%)	2/10 (20%)	0.136
pH	6.5 ± 0.5 (n=26)	5.7 ± 0.5 (n=29)	5.8 ± 0.7 (n=10)	<0.001
Protein (≥15 mg/dL)	4/26 (15.4%)	2/29 (6.9%)	6/10 (60%)	0.001
Specific Gravity	1.013 ± 0.007 (n=26)	1.018 ± 0.007 (n=29)	1.016 ± 0.006 (n=10)	0.053
Urobilinogen (≥2 mg/dL)	0/26 (0%)	2/29 (6.9%)	2/10 (20%)	0.080

Supplement 3

Male Serum Factor	Control Mean ± SD	Type 2 Diabetic Mean ± SD	Diabetic Nephropathy Mean ± SD	p value
ACR	0.6 ± 0.1 (n=6)	0.7 ± 0.2 (n=20)	0.2 ± 0.1 (n=16)	<0.001
Adiponectin (ng/ml)	5466.0 ± 2961.8 (n=4)	3764.7 ± 2450.1 (n=12)	4485.4 ± 2636.3 (n=12)	0.497
Albumin (g/L)	43.4 ± 2.6 (n=6)	44.8 ± 2.0 (n=20)	41.0 ± 3.4 (n=16)	0.002
Creatinine (μmol/L)	73.7 ± 13.0 (n=6)	68.9 ± 14.8 (n=20)	202.3 ± 81.0 (n=16)	<0.001
CRP (ng/ml)	1202.8 ± 906.2 (n=4)	4316.3 ± 4170.2 (n=12)	8264.1 ± 6845.4 (n=12)	0.034
Cystatin C (mg/L)	0.8 ± 0.2 (n=6)	0.9 ± 0.2 (n=20)	2.0 ± 0.6 (n=16)	<0.001
EGF (pg/ml)	85.7 ± 53.9 (n=6)	28.4 ± 28.2 (n=20)	17.2 ± 34.7 (n=16)	0.001
eGFR	98.1 ± 22.7 (n=6)	108.0 ± 31.1 (n=20)	35.3 ± 20.9 (n=16)	<0.001
H-FABP (ng/ml)	4.9 ± 2.4 (n=6)	5.8 ± 2.1 (n=19)	19.2 ± 8.1 (n=16)	<0.001
L-FABP (ng/ml)	1.9 ± 1.9 (n=6)	2.6 ± 3.7 (n=20)	8.7 ± 5.8 (n=16)	0.001
Glucose (mmol/L)		11.4 ± 3.4 (n=19)	11.3 ± 3.7 (n=8)	0.958
HbA1c (mmol/mol)		73.9 ± 10.4 (n=18)	76.7 ± 18.1 (n=10)	0.962
HDL Cholesterol (mmol/L)	1.6 ± 0.4 (n=6)	1.2 ± 0.3 (n=20)	1.1 ± 0.4 (n=16)	0.015
IFN-γ (pg/ml)	0.1 ± 0.1 (n=6)	0.4 ± 0.6 (n=20)	0.4 ± 0.6 (n=16)	0.114
IL-10 (pg/ml)	0.4 ± 0.1 (n=6)	0.6 ± 0.3 (n=20)	0.9 ± 0.5 (n=16)	0.016
IL-1α (pg/ml)	0.1 ± 0.0 (n=6)	0.1 ± 0.1 (n=20)	0.2 ± 0.4 (n=16)	0.209
IL-1β (pg/ml)	1.0 ± 1.1 (n=6)	0.8 ± 0.2 (n=20)	1.7 ± 2.0 (n=16)	0.032
IL-2 (pg/ml)	0.9 ± 1.2 (n=6)	0.7 ± 0.7 (n=20)	0.8 ± 0.9 (n=16)	0.866
IL-4 (pg/ml)	1.5 ± 0.5 (n=6)	1.3 ± 0.4 (n=20)	2.2 ± 4.1 (n=16)	0.207
IL-6 (pg/ml)	0.8 ± 0.5 (n=6)	2.4 ± 1.8 (n=20)	9.3 ± 15.3 (n=16)	<0.001
IL-8 (pg/ml)	4.8 ± 1.1 (n=6)	11.6 ± 9.9 (n=20)	29.5 ± 70.4 (n=16)	0.013
Insulin (pmol/L)	221.7 ± 226.2 (n=6)	137.1 ± 94.2 (n=20)	209.7 ± 179.3 (n=16)	0.613
LDL Cholesterol (mmol/L)	2.9 ± 0.4 (n=6)	2.5 ± 0.7 (n=20)	1.7 ± 0.7 (n=16)	0.002
MCP-1 (pg/ml)	175.6 ± 91.4 (n=6)	209.0 ± 87.4 (n=20)	208.3 ± 84.1 (n=16)	0.810
Midkine (pg/ml)	164.6 ± 206.2 (n=6)	108.7 ± 66.0 (n=20)	2521.7 ± 3289.6 (n=16)	<0.001
MIP-1α (pg/ml)	4.5 ± 1.8 (n=6)	7.0 ± 2.6 (n=20)	10.5 ± 3.6 (n=16)	<0.001
NGAL (ng/ml)	66.3 ± 23.1 (n=4)	49.1 ± 18.5 (n=12)	132.7 ± 90.6 (n=12)	0.004
sTNFR1 (ng/ml)	0.6 ± 0.2 (n=6)	0.7 ± 0.2 (n=20)	2.9 ± 2.0 (n=16)	<0.001
sTNFR2 (ng/ml)	1.2 ± 0.5 (n=6)	1.7 ± 0.7 (n=20)	6.1 ± 3.3 (n=16)	<0.001
TAS (mmol/L)	1.7 ± 0.1 (n=6)	1.9 ± 0.2 (n=20)	1.7 ± 0.3 (n=16)	0.100
TNFα (pg/ml)	2.2 ± 0.6 (n=6)	2.9 ± 1.0 (n=20)	4.6 ± 2.6 (n=16)	<0.001
Total Cholesterol (mmol/L)	5.1 ± 0.4 (n=6)	4.5 ± 1.2 (n=20)	3.5 ± 0.8 (n=16)	0.001
Transferrin (g/L)	2.4 ± 0.4 (n=6)	2.7 ± 0.3 (n=20)	2.2 ± 0.7 (n=16)	0.009
Triglyceride (mmol/L)	1.2 ± 0.4 (n=6)	2.9 ± 2.5 (n=20)	1.8 ± 0.8 (n=16)	0.038
Urea (mmol/L)	6.4 ± 2.1 (n=6)	5.8 ± 1.3 (n=20)	8.3 ± 4.9 (n=16)	0.144
VEGF (pg/ml)	78.6 ± 65.3 (n=6)	84.8 ± 60.1 (n=20)	103.7 ± 96.8 (n=16)	0.983

Female Serum	Control	Type 2 Diabetic	Diabetic Nephropathy	
**Factor**	**Mean ± SD**	**Mean ± SD**	**Mean ± SD**	**p value**
ACR	0.7 ± 0.1 (n=20)	0.8 ± 0.2 (n=11)	0.3 ± 0.2 (n=12)	<0.001
Adiponectin (ng/ml)	9123.3 ± 4009.2 (n=14)	3498.7 ± 1697.5 (n=9)	9215.1 ± 5945.9 (n=8)	0.002
Albumin (g/L)	43.5 ± 1.7 (n=20)	44.3 ± 2.0 (n=12)	41.4 ± 8.8 (n=12)	0.062
Creatinine (μmol/L)	60.2 ± 9.0 (n=20)	56.7 ± 13.4 (n=11)	163.8 ± 93.5 (n=12)	<0.001
CRP (ng/ml)	2812.5 ± 5700.5 (n=14)	5397.6 ± 5277.9 (n=9)	11513.6 ± 8262.9 (n=8)	0.003
Cystatin C (mg/L)	0.7 ± 0.1 (n=20)	0.9 ± 0.2 (n=12)	1.9 ± 0.7 (n=12)	<0.001
EGF (pg/ml)	66.9 ± 45.5 (n=20)	51.0 ± 44.9 (n=11)	26.4 ± 31.0 (n=12)	0.021
eGFR	91.9 ± 17.7 (n=20)	100.0 ± 28.5 (n=11)	38.0 ± 25.2 (n=12)	<0.001
Glucose (mmol/L)		10.9 ± 4.1 (n=11)	14.5 ± 3.9 (n=4)	0.117
HbA1c (mmol/mol)		66.1 ± 13.5 (n=11)	68.8 ± 19.7 (n=10)	0.832
HDL Cholesterol (mmol/L)	1.8 ± 0.4 (n=20)	1.3 ± 0.3 (n=12)	1.3 ± 0.3 (n=12)	<0.001
H-FABP (ng/ml)	4.4 ± 1.9 (n=20)	5.3 ± 2.2 (n=11)	16.2 ± 7.2 (n=12)	<0.001
IFN-ɣ (pg/ml)	4.0 ± 16.3 (n=20)	0.3 ± 0.5 (n=11)	0.6 ± 0.7 (n=12)	0.419
IL-10 (pg/ml)	0.6 ± 0.4 (n=20)	0.5 ± 0.3 (n=11)	0.8 ± 0.7 (n=12)	0.076
IL-1α (pg/ml)	0.2 ± 0.2 (n=20)	0.1 ± 0.0 (n=11)	0.1 ± 0.1 (n=12)	0.097
IL-1β (pg/ml)	1.2 ± 1.0 (n=20)	1.3 ± 0.7 (n=11)	0.7 ± 0.4 (n=12)	0.144
IL-2 (pg/ml)	1.3 ± 1.7 (n=20)	0.6 ± 0.7 (n=11)	0.6 ± 0.6 (n=12)	0.271
IL-4 (pg/ml)	1.9 ± 1.6 (n=20)	1.3 ± 0.3 (n=11)	1.3 ± 0.3 (n=12)	0.008
IL-6 (pg/ml)	1.2 ± 0.9 (n=20)	5.3 ± 11.3 (n=11)	5.0 ± 4.2 (n=12)	0.001
IL-8 (pg/ml)	6.8 ± 3.6 (n=20)	10.7 ± 6.8 (n=11)	11.6 ± 10.4 (n=12)	0.137
Insulin (pmol/L)	120.5 ± 203.4 (n=20)	166.0 ± 119.5 (n=12)	150.1 ± 119.4 (n=12)	0.065
LDL Cholesterol (mmol/L)	2.7 ± 0.9 (n=20)	2.6 ± 0.9 (n=12)	2.4 ± 1.2 (n=12)	0.756
L-FABP (ng/ml)	0.9 ± 1.7 (n=20)	3.4 ± 4.7 (n=12)	6.3 ± 5.8 (n=12)	0.004
MCP-1 (pg/ml)	171.3 ± 70.4 (n=20)	230.7 ± 87.2 (n=11)	214.8 ± 103.2 (n=12)	0.219
Midkine (pg/ml)	67.6 ± 98.2 (n=20)	290.3 ± 687.9 (n=12)	2300.4 ± 3863.8 (n=12)	<0.001
MIP-1α (pg/ml)	5.3 ± 2.0 (n=20)	11.2 ± 8.8 (n=12)	11.2 ± 4.3 (n=12)	<0.001
NGAL (ng/ml)	54.3 ± 16.3 (n=14)	48.9 ± 11.9 (n=9)	148.0 ± 82.5 (n=8)	0.007
sTNFR1 (ng/ml)	0.5 ± 0.1 (n=20)	0.7 ± 0.2 (n=12)	2.4 ± 1.3 (n=12)	<0.001
sTNFR2 (ng/ml)	1.1 ± 0.3 (n=20)	1.7 ± 0.5 (n=12)	7.0 ± 3.8 (n=12)	<0.001
TAS (mmol/L)	1.7 ± 0.2 (n=20)	1.8 ± 0.1 (n=12)	1.9 ± 0.3 (n=12)	0.028
TNFα (pg/ml)	3.9 ± 6.4 (n=20)	2.9 ± 1.2 (n=11)	5.1 ± 2.3 (n=12)	0.001
Total Cholesterol (mmol/L)	5.1 ± 0.9 (n=20)	4.5 ± 0.8 (n=12)	4.4 ± 1.4 (n=12)	0.11
Transferrin (g/L)	2.4 ± 0.3 (n=20)	2.9 ± 0.3 (n=12)	2.2 ± 0.7 (n=12)	0.001
Triglyceride (mmol/L)	1.2 ± 0.6 (n=20)	1.8 ± 0.8 (n=12)	1.7 ± 0.8 (n=12)	0.028
Urea (mmol/L)	5.2 ± 1.0 (n=20)	5.4 ± 1.4 (n=12)	7.6 ± 4.4 (n=12)	0.145
VEGF (pg/ml)	92.4 ± 74.1 (n=20)	63.3 ± 35.2 (n=11)	103.0 ± 137.7 (n=12)	0.688

Male Urine	Control	Type 2 Diabetic	Diabetic Nephropathy	
**Factor**	**Mean ± SD**	**Mean ± SD**	**Mean ± SD**	**p value**
ACR	2.0 ± 3.0 (n=6)	2.6 ± 3.3 (n=19)	20.7 ± 18.7 (n=8)	0.008
Clusterin (ng/ml)	211.2 ± 156.7 (n=6)	106.1 ± 194.8 (n=19)	102.0 ± 89.6 (n=8)	0.077
Creatinine (μmol/L)	9142.1 ± 3269.7 (n=6)	6248.1 ± 2820.4 (n=19)	6222.6 ± 2439.1 (n=8)	0.110
Cystatin C (ng/ml)	17.9 ± 8.8 (n=6)	16.3 ± 13.9 (n=19)	22.4 ± 13.6 (n=8)	0.315
EGF (pg/ml)	516.0 ± 0.0 (n=6)	516.0 ± 0.0 (n=19)	516.0 ± 0.0 (n=8)	NaN
IFN-ɣ (pg/ml)	0.0 ± 0.1 (n=6)	0.1 ± 0.1 (n=19)	0.1 ± 0.2 (n=8)	0.915
IL-10 (pg/ml)	0.4 ± 0.2 (n=6)	0.5 ± 0.1 (n=19)	0.5 ± 0.1 (n=8)	0.414
IL-1α (pg/ml)	0.7 ± 0.7 (n=6)	0.6 ± 1.1 (n=19)	0.6 ± 0.8 (n=8)	0.264
IL-1β (pg/ml)	8.9 ± 12.9 (n=6)	1.5 ± 1.6 (n=19)	4.4 ± 7.3 (n=8)	0.347
IL-2 (pg/ml)	0.6 ± 0.5 (n=6)	0.5 ± 0.5 (n=19)	1.0 ± 0.4 (n=8)	0.114
IL-4 (pg/ml)	2.0 ± 0.3 (n=6)	1.8 ± 0.3 (n=19)	1.6 ± 0.2 (n=8)	0.066
IL-6 (pg/ml)	23.2 ± 42.7 (n=6)	2.3 ± 2.5 (n=19)	1.8 ± 1.8 (n=8)	0.737
IL-8 (pg/ml)	182.5 ± 283.7 (n=6)	34.8 ± 76.9 (n=19)	68.6 ± 134.0 (n=8)	0.139
KIM-1 (pg/ml)	1100.8 ± 618.9 (n=6)	1295.8 ± 1507.9 (n=19)	2239.1 ± 3029.8 (n=8)	0.538
L-FABP (ng/ml)	32.6 ± 18.4 (n=6)	8.7 ± 12.9 (n=19)	28.7 ± 27.5 (n=8)	0.001
MCP-1 (pg/ml)	209.5 ± 280.6 (n=6)	106.5 ± 97.9 (n=19)	76.4 ± 34.6 (n=8)	0.796
Microalbumin (mg/L)	19.4 ± 30.5 (n=6)	16.0 ± 20.6 (n=19)	125.7 ± 108.3 (n=8)	0.038
Midkine (pg/ml)	84.9 ± 15.1 (n=6)	1856.7 ± 3017.2 (n=19)	1476.2 ± 1246.7 (n=8)	0.031
MIP-1α (pg/ml)	18.4 ± 9.2 (n=6)	4.2 ± 6.4 (n=19)	9.9 ± 10.3 (n=8)	0.001
NGAL (ng/ml)	88.3 ± 123.7 (n=6)	36.2 ± 55.1 (n=19)	44.2 ± 30.6 (n=8)	0.132
Osmolality (mOsm)	621.8 ± 245.8 (n=6)	837.5 ± 187.3 (n=19)	548.1 ± 122.4 (n=8)	0.002
Protein (mg/ml)	0.1 ± 0.1 (n=6)	0.1 ± 0.0 (n=19)	0.3 ± 0.2 (n=8)	0.041
sTNFR1 (ng/ml)	1.3 ± 0.8 (n=6)	1.4 ± 0.8 (n=19)	2.3 ± 1.5 (n=8)	0.302
sTNFR2 (ng/ml)	3.2 ± 2.1 (n=6)	4.9 ± 2.9 (n=19)	6.7 ± 4.0 (n=8)	0.196
TNFα (pg/ml)	3.2 ± 1.1 (n=6)	3.3 ± 0.7 (n=19)	3.0 ± 0.6 (n=8)	0.724
VEGF (pg/ml)	149.3 ± 64.7 (n=6)	62.5 ± 70.7 (n=19)	94.4 ± 55.2 (n=8)	0.011

Female Urine	Control	Type 2 Diabetic	Diabetic Nephropathy	
**Factor**	**Mean ± SD**	**Mean ± SD**	**Mean ± SD**	**p value**
ACR	5.0 ± 8.3 (n=20)	2.9 ± 5.7 (n=10)	15.7 ± 7.3 (n=2)	0.048
Clusterin (ng/ml)	150.1 ± 220.8 (n=20)	69.7 ± 73.0 (n=10)	419.3 ± 550.9 (n=2)	0.759
Creatinine (μmol/L)	6143.2 ± 6340.1 (n=20)	6871.9 ± 2616.1 (n=10)	7807.1 ± 7063.3 (n=2)	0.277
Cystatin C (ng/ml)	14.6 ± 23.5 (n=20)	17.0 ± 7.2 (n=10)	70.6 ± 36.2 (n=2)	0.014
EGF (pg/ml)	619.2 ± 461.5 (n=20)	516.0 ± 0.0 (n=10)	1204.2 ± 973.3 (n=2)	0.038
IFN-ɣ (pg/ml)	0.1 ± 0.1 (n=20)	0.0 ± 0.1 (n=10)	0.0 ± 0.0 (n=2)	0.611
IL-10 (pg/ml)	0.4 ± 0.2 (n=20)	0.6 ± 0.1 (n=10)	0.4 ± 0.1 (n=2)	0.028
IL-1α (pg/ml)	8.4 ± 13.9 (n=20)	2.1 ± 1.8 (n=10)	8.6 ± 6.5 (n=2)	0.087
IL-1β (pg/ml)	14.6 ± 54.0 (n=20)	2.1 ± 2.1 (n=10)	40.9 ± 54.8 (n=2)	0.257
IL-2 (pg/ml)	0.5 ± 0.4 (n=20)	0.4 ± 0.5 (n=10)	0.5 ± 0.7 (n=2)	0.730
IL-4 (pg/ml)	1.8 ± 0.3 (n=20)	1.7 ± 0.2 (n=10)	1.9 ± 0.2 (n=2)	0.365
IL-6 (pg/ml)	5.0 ± 15.5 (n=20)	4.5 ± 9.5 (n=10)	7.6 ± 2.4 (n=2)	0.036
IL-8 (pg/ml)	300.1 ± 868.3 (n=20)	57.6 ± 78.2 (n=10)	1122.6 ± 1517.1 (n=2)	0.322
KIM-1 (pg/ml)	1523.5 ± 3524.6 (n=20)	1785.7 ± 1796.1 (n=10)	5850.5 ± 6140.6 (n=2)	0.050
L-FABP (ng/ml)	36.6 ± 13.9 (n=20)	16.2 ± 20.6 (n=10)	31.6 ± 13.4 (n=2)	0.041
MCP-1 (pg/ml)	83.5 ± 98.8 (n=20)	81.7 ± 41.8 (n=10)	81.2 ± 6.7 (n=2)	0.382
Microalbumin (mg/L)	18.8 ± 41.7 (n=20)	12.3 ± 16.0 (n=10)	96.7 ± 54.0 (n=2)	0.084
Midkine (pg/ml)	120.0 ± 272.4 (n=20)	896.8 ± 1284.4 (n=10)	1311.8 ± 288.4 (n=2)	0.186
MIP-1α (pg/ml)	19.9 ± 6.6 (n=20)	8.7 ± 9.4 (n=10)	15.6 ± 3.2 (n=2)	0.018
NGAL (ng/ml)	81.4 ± 203.6 (n=20)	37.9 ± 23.8 (n=10)	178.8 ± 97.9 (n=2)	0.089
Osmolality (mOsm)	412.5 ± 221.4 (n=20)	763.5 ± 243.3 (n=10)	596.0 ± 456.8 (n=2)	0.010
Protein (mg/ml)	0.1 ± 0.1 (n=20)	0.0 ± 0.0 (n=10)	0.4 ± 0.4 (n=2)	0.100
sTNFR1 (ng/ml)	0.7 ± 0.5 (n=20)	1.5 ± 0.7 (n=10)	4.5 ± 3.5 (n=2)	0.001
sTNFR2 (ng/ml)	1.9 ± 2.3 (n=20)	5.0 ± 2.1 (n=10)	10.1 ± 1.6 (n=2)	0.001
TNFα (pg/ml)	3.6 ± 0.8 (n=20)	3.5 ± 0.8 (n=10)	3.6 ± 0.1 (n=2)	0.848
VEGF (pg/ml)	45.4 ± 61.1 (n=20)	31.3 ± 20.1 (n=10)	68.8 ± 59.8 (n=2)	0.566
